# School-based surveillance of acute infectious disease in children: a systematic review

**DOI:** 10.1186/s12879-021-06444-6

**Published:** 2021-08-03

**Authors:** A. L. Donaldson, J. L. Hardstaff, J. P. Harris, R. Vivancos, S. J. O’Brien

**Affiliations:** 1 NIHR Health Protection Research Unit in Gastrointestinal Infections, University of Liverpool, Liverpool, UK; 2 Institute of Population Health Sciences, University of Liverpool, Liverpool, UK; 3 Field Epidemiology Service, Public Health England, Liverpool, UK

**Keywords:** School absence, School attendance registers, Children, Syndromic surveillance, Infectious disease, Outbreaks

## Abstract

**Background:**

Syndromic surveillance systems are an essential component of public health surveillance and can provide timely detection of infectious disease cases and outbreaks. Whilst surveillance systems are generally embedded within healthcare, there is increasing interest in novel data sources for monitoring trends in illness, such as over-the-counter purchases, internet-based health searches and worker absenteeism. This systematic review considers the utility of school attendance registers in the surveillance of infectious disease outbreaks and occurrences amongst children.

**Methods:**

We searched eight databases using key words related to school absence, infectious disease and syndromic surveillance. Studies were limited to those published after 1st January 1995. Studies based in nursery schools or higher education settings were excluded. Article screening was undertaken by two independent reviewers using agreed eligibility criteria. Data extraction was performed using a standardised data extraction form. Outcomes included estimates of absenteeism, correlation with existing surveillance systems and associated lead or lag times.

**Results:**

Fifteen studies met the inclusion criteria, all of which were concerned with the surveillance of influenza. The specificity of absence data varied between all-cause absence, illness absence and syndrome-specific absence. Systems differed in terms of the frequency of data submissions from schools and the level of aggregation of the data. Baseline rates of illness absence varied between 2.3–3.7%, with peak absences ranging between 4.1–9.8%. Syndrome-specific absenteeism had the strongest correlation with other surveillance systems (r = 0.92), with illness absenteeism generating mixed results and all-cause absenteeism performing the least well. A similar pattern of results emerged in terms of lead and lag times, with influenza-like illness (ILI)-specific absence providing a 1–2 week lead time, compared to lag times reported for all-cause absence data and inconsistent results for illness absence data.

**Conclusion:**

Syndrome-specific school absences have potential utility in the syndromic surveillance of influenza, demonstrating good correlation with healthcare surveillance data and a lead time of 1–2 weeks ahead of existing surveillance measures. Further research should consider the utility of school attendance registers for conditions other than influenza, to broaden our understanding of the potential application of this data for infectious disease surveillance in children.

**Systematic review registration:**

PROSPERO 2019 CRD42019119737.

**Supplementary Information:**

The online version contains supplementary material available at 10.1186/s12879-021-06444-6.

## Background

Public Health surveillance is the “continuous, systematic collection, analysis and interpretation of health-related data needed for the planning, implementation, and evaluation of public health practice.” [1] For infectious disease, timely surveillance systems are fundamental to providing early detection of cases and outbreaks, allowing measures to be put in place to protect others and reduce transmission [2]. Historically, public health surveillance was disease-specific, relying on clinical diagnoses and laboratory reports [3]. However, such surveillance systems can be subject to significant delays and over recent years there has been increasing recognition of the value of syndromic surveillance in providing more timely detection of infectious illness [4–7].

Syndromic surveillance can be based on either the identification of clinical syndromes that are indicative of a given disease, or the clustering of non-specific symptoms and changes in patterns of health behaviours which could indicate an outbreak or unusual event [8]. Syndromic surveillance systems have been developed using multiple sources of data, many of which are embedded within healthcare, such as emergency department attendances, ambulance dispatches or calls to remote telehealth services [9–14]. However, there is increasing interest in the use of novel sources of data, such as over-the-counter purchases, internet-based health searches and worker absenteeism, which have been found to correlate well with traditional surveillance measures [11, 15–21].

School attendance registers offer a novel dataset which could be used to provide more timely information regarding infectious disease and outbreaks amongst children [22]. Children are commonly affected by gastrointestinal illness and respiratory illness, both of which are key causes of illness absence from school [23–26]. Children are recognised as important transmitters of infection, [27–30] and schools are principal settings in the spread of infections between children [29, 31]. Close household contact with parents and grandparents facilitates the spread of illness from schools into the wider community [32, 33]. School absence data could support the early identification of outbreaks within schools, enabling timely intervention to reduce the transmission of infections both within and outside of the school setting. Furthermore, as school absence may occur from the first day of illness, this novel dataset has the potential to offer more timely data than healthcare-based surveillance. There is evidence that children may be the first affected by seasonal and pandemic illnesses, [34–36] and by enhancing the detection of disease in children such data could provide early warning of infections before they start circulating in the wider community.

This systematic review considers the utility of school attendance registers in the surveillance of infectious disease outbreaks and occurrences amongst children. The value of a school-based surveillance system will be considered in terms of its correlation and lead time compared to traditional surveillance measures. A secondary objective of this review is to describe the burden of illness absenteeism and outbreaks in school-aged children.

## Methods

### Protocol and registration

The systematic review protocol was registered on PROSPERO in January 2019 (PROSPERO 2019 CRD42019119737) [37]. The protocol and article follow the PRISMA checklist for the reporting of systematic reviews.

### Eligibility criteria

The population of interest for this review was children aged between 4 and 18 years, attending school. Only studies published on or after 1st January 1995 and available in English were included. As this review considers what school attendance data adds to existing health surveillance systems, studies were limited to those from OECD countries, [38] which are likely to have established health surveillance systems in place for comparison. No limitation was put on school type, but studies based in nursery schools or higher education settings were excluded, as these settings are not components of compulsory education and may be subject to different requirements for attendance and absence reporting. Review papers, editorials, book chapters, conference abstracts or proceedings, randomised controlled trails and case reports were also excluded. Following the full text review of articles, qualitative studies and statistical papers exploring novel mathematical techniques to modelling disease surveillance data were added to the excluded study types.

### Information sources

The following electronic databases were searched: Medline, Web of Science, Pubmed, Scopus, Science Direct, Biosis Previews, Open Grey and Proquest dissertations and theses. The searches were conducted on 23 October 2018.

### Search terms

The following search terms were used: ((population surveillance/ or public health surveillance/ or sentinel surveillance/ or surveillance .mp.) OR (syndromic surveillance.mp.) OR (attend*.mp.) OR (absenteeism/ or absen*.mp.) OR (registers.mp.)) AND ((school.mp. or Schools/) OR (school aged children.mp.) OR (school children.mp.)) AND ((Infectious disease.mp. or Communicable Diseases/) OR (Outbreaks.mp. or Disease Outbreaks/) OR (epidemics.mp. or EPIDEMICS/) OR (pandemics.mp. or PANDEMICS/) OR (bugs.mp.)). The search terms were piloted before use and combined using Boolean operators. The search terms were developed for use in MEDLINE. Where possible, the same terms were used in each database, but some adaptation or simplification was required to meet the search requirements of different databases (Additional file 1). The terms were searched for within the title and abstracts of papers and, where possible, the keywords.

### Study selection

References from each database were imported into Mendeley reference manager. Each reference list was first de-duplicated, before combining all references and conducting a further removal of duplicate references. Additional duplicates were removed by manual searching. Two independent reviewers (AD and JLH) then undertook screening of the article titles and abstracts, applying the agreed exclusion and inclusion criteria. Any discrepancies between the reviewers were discussed and consensus reached. Articles meeting the screening criteria underwent a full text review. This was conducted by two reviewers (AD and JPH) using agreed eligibility criteria. Consensus was reached between the reviewers about the final articles for inclusion. Reference lists of the included articles were searched to identify any additional relevant studies not identified as part of the original search strategy. Papers identified in this way underwent the same screening and full text review outlined above

### Data collection process and data items

Data extraction was performed using a standardised data extraction form (Additional file 2). Where available, the following data items were extracted; year of publication, country, prospective or retrospective study, age group, school type, sample size, time period of data collection, organism/syndrome, purpose of surveillance (case ascertainment or outbreak detection), case or outbreak definition, primary outcome measure, description of surveillance system (including the specificity, timeliness and spatial-temporal level of data collected), comparator surveillance systems, absenteeism rates with 95% confidence intervals, correlation measures with *p*-values, and lead or lag times compared to other surveillance measures.

### Summary measures

The summary measures were descriptive of the school surveillance systems and the methods used within each study. Outcomes included estimates of absenteeism, correlation measures and lead/lag times. Due to a high level of heterogeneity, estimates could not be pooled between studies.

### Synthesis of results

A narrative synthesis approach was adopted, comparing and contrasting the school-based surveillance systems in terms of their design, purpose, population, and performance against existing health surveillance systems.

## Results

### Study selection

**Fig. 1:**
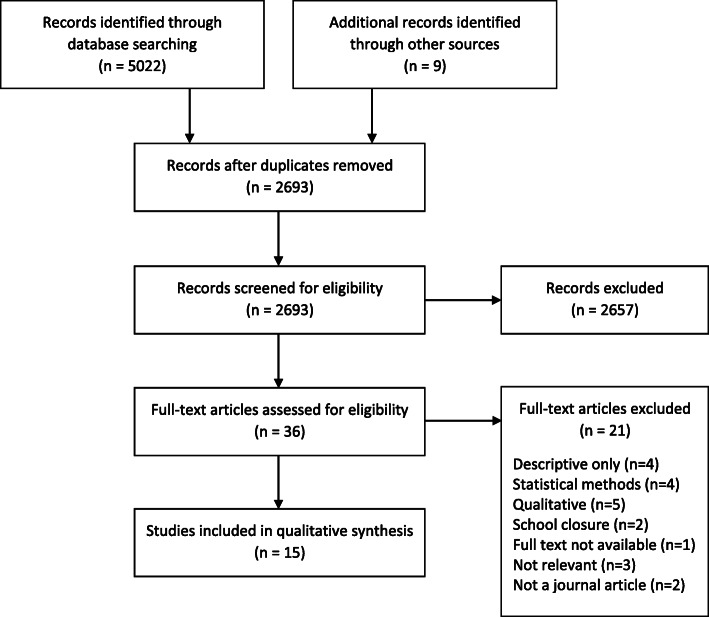
Flow diagram of study selection

The initial searches identified 5022 references, which reduced to 2684 once duplicates were removed. After screening the abstracts, 33 studies met the eligibility criteria for full text review. Of these, 14 were included in the systematic review. Nine additional studies were identified through searching the references of the papers for inclusion. Following abstract screening, three underwent full text review, one of which was subsequently included in the systematic review, giving a total of 15 studies (Fig. 1).

### Characteristics of included studies

**Fig. 2:**
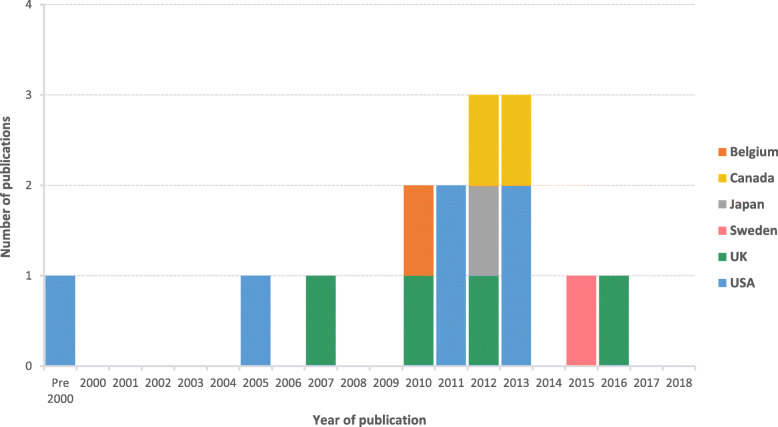
Year and country of publication of included studies. *Studies published pre-2000 comprised of one study published in 1995

All of the studies identified were concerned with the surveillance of influenza and over half were related to pandemic influenza. This is reflected in a peak of studies published between 2010 and 2013, following the H1N1 pandemic in 2009 (Fig. 2). The greatest number of studies identified originated from the USA (*n* = 6), with multiple studies also reported from the UK (*n* = 4) and Canada (*n* = 2).

A summary of the included studies is outlined in Table 1. Over half (9/15 studies) collected prospective data, the majority of which were during the H1N1 pandemic. Sample size varied from six schools to over 3000 schools. Most studies included data on all school age groups, ranging from 3 to 18 years of age.

**Table 1:** Description of included studies

Author	Year of Publication	Country	Organism/ syndrome	Prospective or retrospective	School ages	Sample size	Specificity of absence	Frequency of data submissions
Aldridge et al [51]	2016	UK	Seasonal influenza	Prospective	11-16 yrs	27 schools	Medical ^a^	Weekly
Besculides et al [39]	2005	USA	Seasonal influenza	Retrospective	5-18 yrs	1160 schools	All cause	Daily
Bollaerts et al. [21]	2010	Belgium	Pandemic influenza	Prospective	3-18 yrs	~ 1 million pupils	Illness	Weekly
Chu et al [52]	2013	Canada	Pandemic influenza	Retrospective	4-14 yrs	8 PHUs ^b^	Variable	Not specified
Crawford et al [40]	2011	USA	Pandemic influenza	Prospective	5-12 yrs	80 schools	All cause	Daily
Kara et al [43]	2012	UK	Pandemic influenza	Retrospective	4-18 yrs	373 schools	Illness	Weekly
Kightlinger et al [44]	2013	USA	Pandemic influenza	Prospective	5-18 yrs	187 schools	Illness	Weekly
Kom Mogto et al [49]	2012	Canada	Pandemic influenza	Prospective	6-17 yrs	3432 schools	Syndrome-specific	Daily
Lenaway et al [45]	1995	USA	Seasonal influenza	Prospective	5-18 yrs	44 schools	Illness	Weekly
Ma et al [46]	2015	Sweden	Seasonal influenza	Retrospective	6-16 yrs. ^c^	500 schools	Illness	Not specified
Mann et al [41]	2011	USA	Pandemic influenza	Prospective	5-18 yrs	349 schools	All cause	Daily
Mook et al [47]	2007	UK	Seasonal influenza	Prospective	4-16 yrs	11 schools	Illness	Daily
Schmidt et al [48]	2010	UK	Seasonal influenza	Retrospective	5-11 yrs	6 schools	Illness	Not specified
Suzue et al [50]	2012	Japan	Pandemic influenza	Retrospective	3-18 yrs	142 schools	Syndrome specific	Daily
Williams et al. [42]	2013	USA	Pandemic influenza	Prospective	5-17 yrs	216 schools	All cause + syndrome specific	Weekly

^a^ Medical absences include illness absence and absence to attend medical appointments

^b^ Public Health Units (PHUs) varied in size and were broadly divided into large PHUs (population > 400,000) and small PHUs (population ≤ 400,000). Each PHU had a custom surveillance system to measure school absenteeism, collecting data on all cause absenteeism (8 PHUs), illness absenteeism (1 PHU) and respiratory illness absence (1 PHU) from schools within their area

^c^ Not clearly specified. The school ages noted are for compulsory education in the country of study

### Description of methods used for school-based surveillance

The three most common forms of absence data were all-cause absenteeism, [39–42] illness absenteeism, [21, 43–48] and syndrome-specific absenteeism, which in these studies corresponded to influenza-like-illness (ILI) absences [42, 49, 50]. One paper reported medical absences, which combined both illness and planned medical appointments [51]. Another reported data from across multiple health authorities, each of which had a different system in place, varying between all-cause absence, illness absence and respiratory absence [52].

The frequency of data submissions from schools varied between daily [39–41, 47, 49, 50] and weekly reports [21, 42–45, 51]. Weekly reports often contained details of daily absences, so the frequency of reporting did not necessarily affect the level to which the data were analysed. Most studies analysed either daily or weekly absence rates but five studies used exceedances over a threshold as an indicator of a suspected outbreak [41, 42, 45, 49, 52]. One additional study used an absence threshold at city-level to determine the beginning and end points of the H1N1 influenza pandemic [50]. Outbreak definitions varied and are detailed in Table 2.

**Table 2:** Outbreak definitions used within included studies

First Author & year of publication	Outbreak threshold / alert	Time period of breach
Chu 2013 [52]	Exceedance based on C2-MEDIUM method^a^ OR > 5% all-cause absenteeism^b^	Single day Single day
Kom Mogto 2012 [49]	≥10% ILI-related absenteeism	Single day
Lenaway 1995 [45]	> 7.5% illness absence	Single week average
Mann 2011 [41]	≥8% all-cause absenteeism AND 1 SD above 30 day mean	Single day
Suzue 2012^c^ [50]	> 2% ILI-related absenteeism	Single day
Williams 2013 [42]	> 10% all-cause absenteeism > 5% ILI-related absenteeism	2 or more consecutive school days

^a^ C2-MEDIUM method calculates the mean and standard deviation (SD) from −9 to −3 days before the day of interest. Threshold is an exceedance of the expected value by three standard deviations

^b^ Not clearly stated, assumed from description of methods

^c^ Threshold used to detect start and end of pandemic

The majority of studies aggregated absences across geographical areas or groups of schools, with only five studies considering absences at the individual school-level [41, 42, 45, 48, 49].

### Estimates of the burden of absenteeism

**Fig. 3:**
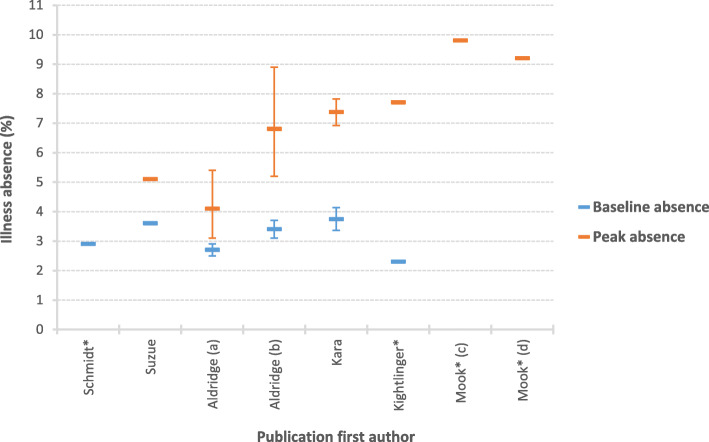
Percentage of illness absenteeism at baseline and peak during influenza season or outbreak, with 95% confidence intervals. *No published confidence interval; (**A**) 2011/12 estimate; (**B**) 2012/13 estimate; (**C**) 4–11 year olds; (**D**) 11–16 year olds

No standard measure of absenteeism was used across the included studies. Therefore, we were unable to conduct a pooled estimate of the impact of illness or influenza on school absences. Studies reported a mix of baseline absences, peak absences or both, either aggregated across all school age groups or by school type. Six studies reported rates of illness absenteeism (Fig. 3). Baseline illness absences varied from 2.3–3.7%, [43, 44, 48, 50, 51] with peak illness absence ranging from 4.1 to 9.8% [43, 44, 47, 50, 51]. Two studies reported all-cause absenteeism rates, with results varying from 4.4–17.8% [39, 40]. The higher reported values were for older children aged 14-18 yrs. Four papers did not directly report on either the percentage of absenteeism or the number of exceedances identified, but instead reported only trends or correlations [21, 42, 45, 46].

### Estimates of correlation with health surveillance measures

The majority of studies used laboratory isolates as the conclusive marker of influenza activity. Other surveillance measures used for comparison included primary care consultations, hospitalisations or emergency department attendances, telehealth calls and death certifications. The relationship between school absenteeism and established surveillance measures was investigated by study authors using visual inspection and correlation coefficients. The measures of correlation varied and as a result it was not possible to generate a pooled estimate. Tests of correlation included Spearman Rank, Pearson’s r, cross-correlation analysis and the coefficient of determination.

The correlation between all-cause absenteeism and other surveillance measures were explored in four studies. Whilst visual inspection suggested that peaks in all-cause absenteeism coincided with community outbreaks, [39] correlations between laboratory reports and both all-cause absences and outbreaks based on > 10% all-cause absence were low (r = 0.33^1^ and r = 0.39* respectively, *n* = 216 schools) [42]. In a study of 80 schools, all-cause absenteeism was not correlated with ILI emergency department visits during periods of low influenza activity (r_s_ = 0.23, *p* = 0.16), but there was evidence of correlation during periods of high influenza activity (r_s_ = 0.98, *p* = 0.05) [40]. A study of outbreak alerts, based on all-cause absenteeism at 349 schools, generated a high number of alerts (one quarter of schools over a 6 week period), only 10% of which were subsequently confirmed as influenza [41].

Two studies explored the correlation between syndrome-specific absenteeism and other surveillance measures. Based on data from over 3400 schools, strong correlations were reported between the number of schools who reported > 10% ILI-related absence and both laboratory isolates and influenza hospitalisations (r_s_ = 0.90, *p* < 0.02 and r_s_ = 0.83, *p* = 0.01 respectively) [49]. Amongst a smaller number of schools (*n* = 216), there was evidence of correlation between the lower threshold of > 5% ILI-related absence and laboratory isolates, but the correlation coefficient was reduced (r = 0.78*) [42]. The highest reported correlations were between laboratory isolates and ILI-absence rates (r = 0.92*), which increased when ILI absences were shifted back by one week (r = 0.97*), suggesting that trends in ILI absences preceded laboratory reports by one week [42].

Studies exploring illness absenteeism presented mixed results. Based on visual inspection, study authors concluded that the peaks of illness absenteeism preceded or were concurrent with peaks in other surveillance systems across influenza seasons [21, 45, 47]. However, correlation with laboratory data varied between no correlation (*n* = 373 schools), [43] mild to moderate correlation (r = 0.11–0.45*and cross-correlation = 0.52, *p* < 0.001, *n* = 500 and 6 schools respectively), [46, 48] and strong correlation (r = 0.9, *p* < 0.01, *n* = 187 schools) [44]. The study of 187 schools also reported correlations with ILI hospitalisations (r = 0.9, *p* < 0.01) and ILI-related deaths (r = 0.7, *p* < 0.01) [44]. Associations with primary care data ranged from moderate positive correlations to negative correlations (r = − 0.19 – 0.47,^2^*n* = 373–500 schools), [43, 46] and no association was found with telehealth calls [43]. In a study exploring absences at 27 schools, linear regression modelling identified statistically significant associations between medical absences, which include planned appointments, and both primary care data and laboratory reports (r^2^ = 0.42, *p* < 0.001 and r^2^ = 0.27, *p* < 0.001 respectively) [51]. The association with primary care ILI reports was strengthened when this surveillance measure was limited to children aged 5–14 (r^2^ = 0.62, *p* < 0.001).

### Lead and lag times

Thirteen studies considered the lead or lag time of school absence data compared to other surveillance measures. All-cause absenteeism was not found to contribute significantly in terms of timeliness, with the majority of peaks occurring after other surveillance systems, [39, 52] and multiple peaks observed which were unrelated to influenza activity [42].

Illness absence presented a mixed picture, with the timeliness of peaks varying between no lead or lag time, [43, 45, 47, 48] a 1–4 week lead time, [21, 45–47, 51] and a lag time of 1–11 weeks [44, 46]. Syndrome-specific absences peaked concurrently or 1–2 weeks before other surveillance measures, [42, 49] and provided lead time on the start, peak and end point of the H1N1 pandemic (5 day, 10 day and 17 day lead time respectively) [50].

## Discussion

This systematic review identified fifteen papers which explored the utility of school attendance registers in the syndromic surveillance of infectious disease. All of the papers identified were concerned with influenza, either pandemic or seasonal. There was a particular cluster of papers published following the 2009 H1N1 pandemic, indicating the heightened need for community-based surveillance systems during the pandemic. None of the papers we identified considered other common infectious diseases, such as diarrhoea and vomiting.

The specificity of the data collected varied between all-cause absenteeism, illness absenteeism and syndrome-specific (in this case ILI) absenteeism. Syndrome-specific absenteeism had the strongest correlation with other surveillance systems, with illness absenteeism generating mixed results and all-cause absenteeism performing the least well. A similar pattern of results emerged in terms of lead and lag times, with ILI-specific absence providing a 1–2 week lead time, compared to lag times reported for all-cause absence data and inconsistent results for illness absence data. These results would indicate a potential role for syndrome-specific absences in the surveillance of influenza. However, all three studies which utilised syndrome-specific absence were conducted during the H1N1 pandemic, and therefore the results presented may not reflect the performance of these data in non-pandemic situations. It should also be considered whether a two week lead time is sufficient warning to allow additional protective measures to be put in place.

The three studies which used syndrome-specific data also utilised absence thresholds, which were used to trigger alerts at the individual school level. The thresholds used were > 2, > 5% and ≥ 10% ILI-related absenteeism. Whilst the ≥10% threshold provided the strongest correlation with other surveillance measures, it provided less lead time than the > 5 and > 2% thresholds. The scarcity of papers in this area makes it difficult to explore this further, but such thresholds inevitably result in a trade-off between accuracy and timeliness. Absence thresholds may also be influenced by health protection strategies targeted at children, such as vaccination schemes. Such interventions would be expected to reduce peak absence rates and consequently lower thresholds may be required to trigger alerts.

The development of absence thresholds requires an understanding of baseline rates of absence and these have been found to vary by age group. All-cause absenteeism was highest in older children, which could represent absences from causes other than illness. In contrast, both illness absence and symptom-specific absence appeared higher in younger children [43, 47, 49]. There was some indication that influenza started and peaked earlier in younger children, [43, 47] with high schools being affected later [50]. This is consistent with evidence that young children may be the first affected by seasonal and pandemic diseases, [34–36] and highlights the potential value in monitoring infectious illness in elementary/primary school children as an early warning of circulating infections.

As the potential lead time of school absence data was 1–2 weeks, the frequency of data submissions from schools is important in ensuring the early warning is optimised. Whilst the frequency of data reports from schools did not appear to affect correlation with other surveillance systems, the reported 5 day lead time on the start of the H1N1 pandemic may not have provided advanced warning had the data been transferred weekly as oppose to daily. If absence data were utilised to detect and manage outbreaks at the individual school level, daily data submissions would confer additional benefit over weekly reports and aid in the more timely management of localised outbreaks.

### Limitations of school absence data

In the studies identified there was variation in the type of school data used, both between countries and across different health authorities within countries. This makes aggregation of absence data across large areas difficult, [53] and could limit the utility of such data at a national level. School holidays result in a natural break in school attendance data, which is problematic for its use in tracking ongoing community outbreaks. There are also multiple factors which can affect school attendance, making its use in surveillance challenging. All-cause absences will not only capture illness but also unauthorised absences, and has been shown to increase around school holidays [39]. Illness absence will be affected by other infections, such as diarrhoea and vomiting, and has also been found to vary by day of the week [48]. This may contribute to the lack of correlation observed with all-cause and illness absence data, especially during periods of low influenza activity. Increases in school absences may also be affected by media coverage of pandemics or high profile deaths in children, [43, 52] potentially driven by parental concerns of children catching illnesses at school, or lowering their threshold for keeping a child at home if they are unwell. Whilst this is more likely to be an issue in pandemic influenza, which receives significant media coverage, any high profile outbreak is likely to create the same effect, regardless of the underlying organism.

## Conclusion

The evidence of the utility of school attendance registers in the surveillance of infectious illness in children is limited to studies concerned with influenza. Therefore, the findings of this review may not be applicable to other conditions, such as diarrhoea and vomiting. There is a high level of heterogeneity between studies, making it impractical to pool results and generate a meaningful estimate of either burden of illness absenteeism or its correlation with other surveillance measures. However, the studies identified suggest good correlation between syndrome-specific absences and healthcare surveillance data, with a potential lead time especially from absences in younger school age groups. Further research should consider the utility of school attendance registers for conditions other than influenza, to broaden our understanding of the potential application of this data for infectious disease surveillance in children.

## Supplementary Information

**Additional file 1. **Search terms by database. Details of search terms used by database

**Additional file 2. **Data extraction form. Standardised form used in the data extraction process

## Data Availability

The datasets used and/or analysed during the current study are available from the corresponding author on reasonable request.

## Abbreviations

ILI: Influenza-like-illness
OECD: The Organisation for Economic Co-operation and Development
SD: Standard deviation

## Footnotes

1 *P*-value not reported within study paper

2 *P*-value not reported within study paper
