# Supplementary material for: School-based surveillance of acute infectious disease in children: a systematic review
Source: BMC Infect Dis. 2021 Aug 3;21:744. doi: 10.1186/s12879-021-06444-6 (PMC8330200; doi:10.1186/s12879-021-06444-6)
Supplement: Supplementary file 1 — Additional file 1. Search terms by database. Details of search terms used by database [file 12879_2021_6444_MOESM1_ESM.docx]

Search terms by database

**OVID MEDLINE**(R) <1946 to Present>

1. population surveillance/ or public health surveillance/ or sentinel surveillance/ or surveillance .mp.
2. syndromic surveillance.mp.
3. attend*.mp.
4. Absenteeism/ or absen*.mp.
5. registers.mp.
6. 1 or 2 or 3 or 4 or 5
7. school.mp. or Schools/
8. school aged children.mp.
9. school children.mp.
10. 7 or 8 or 9
11. Infectious disease.mp. or Communicable Diseases/
12. Outbreaks.mp. or Disease Outbreaks/
13. epidemics.mp. or EPIDEMICS/
14. pandemics.mp. or PANDEMICS/
15. bugs.mp.
16. 11 or 12 or 13 or 14 or 15
17. 6 and 10 and 16

**Web of Science**

1. **TOPIC:** (surveillance OR "population surveillance" OR "public health surveillance" OR "sentinel surveillance" OR "syndromic surveillance" OR attend* OR absen* OR register)
2. **TOPIC:** (school* OR "school*children" OR "school*aged children")
3. **TOPIC:** ("infectious disease" OR "communicable disease" OR outbreak* OR epidemic* OR pandemic* OR bug*)
4. #1 AND #2 AND #3

**Pubmed**

1. (("surveillance"[Title/Abstract]) OR "attendance"[Title/Abstract]) OR "absenteeism"[Title/Abstract]) OR "register"[Title/Abstract]
2. (("school"[Title/Abstract]) OR "school children"[Title/Abstract]) OR "school aged children"[Title/Abstract]
3. ((((("infectious disease"[Title/Abstract]) OR "communicable disease"[Title/Abstract]) OR "outbreak"[Title/Abstract]) OR "pandemic"[Title/Abstract]) OR "epidemic"[Title/Abstract]) OR "bugs"[Title/Abstract]
4. #1 AND #2 AND #3

**Scopus**

1. ABS ( surveillance OR attendance OR absenteeism OR register )
2. ABS ( school* OR "school*children" OR "school*aged children" )
3. ABS ( "infectious disease" OR "communicable disease" OR outbreak* OR epidemic* OR pandemic* OR bug* )
4. #1 AND #2 AND #3

**Science Direct^[[1]](#footnote-1)^**

1. (surveillance OR attendance OR absenteeism OR register) AND (school) AND ( "infectious disease" OR "communicable disease" OR outbreak OR pandemic)

**Biosis Previews**

1. **TOPIC:** (surveillance OR "population surveillance" OR "public health surveillance" OR "sentinel surveillance" OR "syndromic surveillance" OR attend* OR absen* OR register)
2. **TOPIC:** (school* OR "school*children" OR "school*aged children")
3. **TOPIC:** ("infectious disease" OR "communicable disease" OR outbreak* OR epidemic* OR pandemic* OR bug*)
4. #1 AND #2 AND #3

**Open Grey**

1. Health surveillance AND school

**ProQuest**

1. Ab(surveillance) AND ab(school) AND ab(disease outbreaks)

1. Limited to 8 Boolean operators. Terms searched for within title, abstract and keywords [↑](#footnote-ref-1)
