# Supplementary material for: School-based surveillance of acute infectious disease in children: a systematic review
Source: BMC Infect Dis. 2021 Aug 3;21:744. doi: 10.1186/s12879-021-06444-6 (PMC8330200; doi:10.1186/s12879-021-06444-6)
Supplement: Supplementary file 2 — Additional file 2. Data extraction form. Standardised form used in the data extraction process [file 12879_2021_6444_MOESM2_ESM.docx]

Data extraction form

| **Background information** | |
| --- | --- |
| Article title |  |
| Authors |  |
| Journal |  |
| Year of publication |  |
| **Methods** | |
| Country (+/- area) |  |
| Prospective or retrospective |  |
| Age group(s) |  |
| School type |  |
| Sample size |  |
| Period of data collection |  |
| Organism / syndrome |  |
| Purpose of system (case or outbreak ascertainment, pandemic or seasonal trends) |  |
| Case/absence definition (i.e. missed class, half day, full day) |  |
| Outbreak definition / absence threshold (if applicable) |  |
| Primary outcome measure |  |
| School-level data collected   - Numerator - Denominator |  |
| Specificity of data recorded (all-cause, illness, syndrome specific, micro confirmed) |  |
| Timeliness of data reporting from schools |  |
| Spatial and temporal aggregation of data |  |
| Other surveillance systems / data used |  |
| Methods of data analysis |  |
|  | |
|  | |
|  | |
| **Findings** | |
| Absence rates (+ 95% CI) |  |
| Correlation with other surveillance measures (+ p-values) |  |
| Lead / lag time compared to other surveillance |  |
| Sensitivity of case/outbreak detection |  |
